# Basal and Bolus Insulin Distribution According to Treatment Modality: Data from SWEET Diabetes Registry

**DOI:** 10.1155/2023/8837506

**Published:** 2023-08-09

**Authors:** Ferda Evin, Sascha R. Tittel, Barbara Piccini, Roque Cardona-Hernandez, Dick Mul, Nicole Sheanon, Thekla von dem Berge, Vit Neuman, Martin Tauschmann, Damla Gökşen

**Affiliations:** ^1^Division of Pediatric Endocrinology, Department of Pediatrics, School of Medicine, Ege University, Izmir, Turkey; ^2^Institute of Epidemiology and Medical Biometry, Central Institute for Biomedical Technology (ZIBMT), Ulm University, Ulm, Germany; ^3^German Center for Diabetes Research (DZD), Munich, Neuherberg, Germany; ^4^Diabetology Unit, Meyer University Children's Hospital IRCCS, Florence, Italy; ^5^Division of Pediatric Endocrinology, Hospital Sant Joan de Déu, Barcelona, Spain; ^6^Diabeter, Center for Pediatric and Adult Diabetes Care and Research, Rotterdam, Netherlands; ^7^Division of Pediatric Endocrinology, Department of Pediatrics, University of Cincinnati College of Medicine, Cincinnati, OH, USA; ^8^Kinder und Jugendkrankenhaus AUF DER BULT, Diabetes Center for Children and Adolescents, Hannover, Germany; ^9^Department of Pediatrics, 2nd Faculty of Medicine, Charles University and Motol University Hospital, Prague, Czech Republic; ^10^Department of Pediatrics and Adolescent Medicine, Medical University of Vienna, Vienna, Austria

## Abstract

**Background and Aims:**

The optimal basal and bolus insulin distribution in type 1 diabetes (T1D) is still controversial. Herein, we aimed to determine the variability of basal to total daily insulin dose according to treatment modality and diabetes technologies from the Better Control in Pediatric and Adolescent Diabetes: *Working to Create Centers of Reference* (*SWEET*) *registry. Methods*. The study cohort was generated by using the SWEET database. Patients with T1D for at least 2 years, aged between 2.5 and 18 years, with at least one clinic visit between June 2010 and June 2021, were included in the study. Four groups were composed according to treatment modality as follows: multiple daily injections (MDI) without continuous glucose monitoring (CGM); MDI with CGM; subcutaneous insulin infusion (CSII) without CGM; and CSII with CGM. Data of the participants were analyzed and compared for each treatment modality separately.

**Results:**

A total of 38,956 children and adolescents were included in the study. Of the study sample, 48.6% were female, the median (range) age was 15.2 (11.9–17.2) years, and the median diabetes duration was 6.0 (3.8–9.0) years. The distribution of treatment modality was as follows: MDI without CGM, 32.9%; MDI with CGM, 18.0%; CSII without CGM, 11.7%; and CSII with CGM, 37.3%. In unadjusted data, regardless of treatment modality, all the analyses revealed a significant association between basal dose to total daily insulin dose (BD/TDD) with male gender, younger age group, and lower HbA1c, which were all related to a decreased ratio of BD/TDD (all *p* < 0.05). There was no association between BD/TDD and different diabetes technologies after the age, gender, and diabetes duration were adjusted.

**Conclusions:**

Herein, we showed that there was an association between lower proportions of basal to total insulin and lower hemoglobin A1c in a large cross-sectional cohort of children who had T1D. There was also an association between lower BD/TDD and younger age. There was no significant difference between BD/TDD ratios under different diabetes technologies (CGM and/or CSII).

## 1. Introduction

The primary treatment goal is to maintain near-normoglycemia through intensive insulin therapy, avoid acute complications, and prevent long-term microvascular and macrovascular complications in children and adolescents with type 1 diabetes (T1D).

Modern rapid-acting analogs have not yet defined the optimal percentage of daily basal insulin dose (BD) to total daily insulin dose (TDD) (BD/TDD). The proportion of the total daily dose that has been shown to more effectively attain a lower hemoglobin A1c (HbA1c) is 40%–50% of TDD delivered as basal insulin for the multiple daily injections (MDI) [4]. Further, the same recommendation is made for continuous subcutaneous insulin infusion (CSII) systems that provide basal delivery with short-acting insulin [2]. Lower BD/TDD was associated with lower HbA1c in children and young adults [3], while a multicenter study showed that metabolic control was significantly better in children with a BD/TDD below 0.5 [1].

*The International Society for Pediatric and Adolescent Diabetes* (*ISPAD*) recommends a BD/TDD of 30%–50% [2]. ADA guidelines do not suggest a specific basal insulin percentage [4]. The optimal BD/TDD is still unidentified, and clinical use of BD/TDD still varies, with BD/TDD up to 95% [3, 5]; these high basal ratios are most probably seen in noncompliant individuals with T1D.

In this study, we aimed to examine the followings: (1) to determine the variability of BD/TDD ratios according to age groups, insulin treatment modality, and diabetes technologies; and (2) to determine the association of BD/TDD ratio with glycemic outcome and body mass index (BMI) from the Better Control in Pediatric and Adolescent Diabetes: *Working to Create Centers of Reference* (*SWEET*) *registry*.

## 2. Methods

### 2.1. SWEET

SWEET (https://www.sweetproject.eu) is a network consisting of multinational healthcare centers for diabetic children, adolescents, and young adults. The main mission of SWEET is to provide optimal clinical outcomes and standards for pediatric diabetes worldwide (12). The centers included in the SWEET registry system submit main standardized data to the Institute of Epidemiology and Medical Biometry, Ulm University, Ulm, Germany, bi-annually, either through the Diabetes-Patienten-Verlaufsdokumentation (DPV)-for-SWEET software (https://sweet.zibmt.uni-ulm.de/software.php) developed at Ulm University, from national registries, or through local clinical electronic health records. A team at Ulm University evaluates the validity of the data plausibility and the presence of inconsistent or missing data. Accordingly, a correction request is sent to the corresponding submitting center. All centers contributing data must comply with current regulatory data security and ethics. In total, 129 SWEET centers are currently present in five geographic hubs; Europe, Asia/Middle East/Africa, Australia/New Zealand, North America, and South America.

### 2.2. Selection Data and Study Population

**Figure 1:**
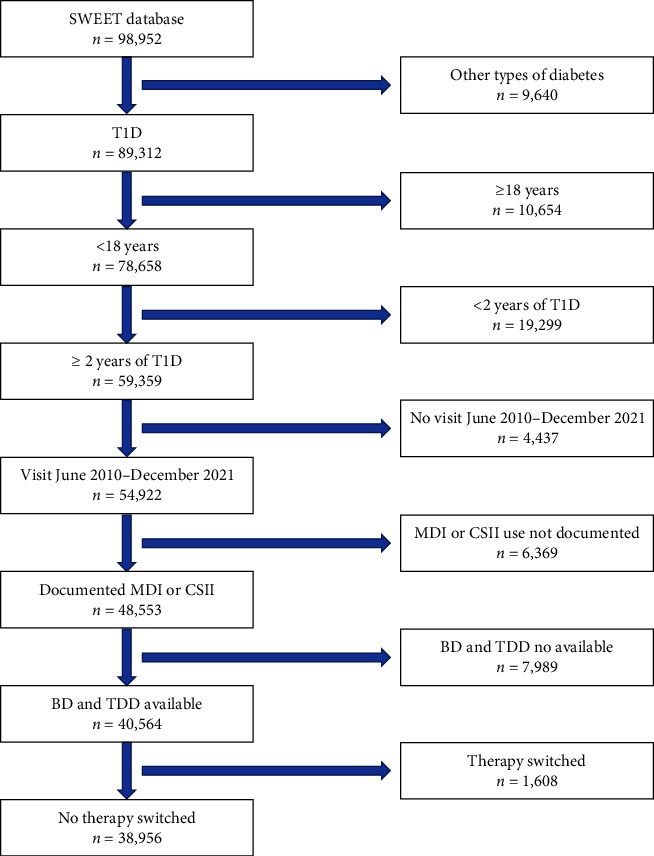
Flowchart diagram of the patient selection during the study process.

Individuals were selected for analysis if they fulfilled the following criteria: (1) T1D; (2) aged 2.5–<18 years; (3) ≥2 years diabetes duration; (4) at least one clinical visit between June 2010 and December 2021; (5) documented MDI or CSII therapy with basal and bolus doses available; and (6) no therapy switch in the individuals' most recent treatment year.  Figure 1 shows a flowchart diagram of the patient selection during the study process.

### 2.3. Research Design and Collected Data

Age, gender, diabetes duration, age of the diabetes onset, daily total insulin dose per body weight in kilograms, the type of administered insulin (MDI or CSII), type of glucose monitoring, sensor use, HbA1c level, the number of severe hypoglycemia (SH) and diabetic ketoacidosis (DKA) attacks were recorded in the individuals' respective most recent treatment year.

BMI was calculated by using the standard formula; weight in kilograms/(height in meters)^2^ (kg/m^2^), and converted to BMI-SDS via World Health Organization charts as reference [6, 7]. BMI classification was evaluated as follows; BMI-SDS ≤−2 SD (underweight), −2 <SD BMI-SDS ≤+1 SD (normal weight), 1 SD <BMI-SDS ≤2 SD (overweight), and BMI-SDS >2 SD (obese).

HbA1c was measured locally and standardized to the diabetes control and complications trial reference of 4%–6% (20–42 mmol/mol) (1,15). HbA1c <7.0% is classified as HbA1c in the target range [8].

Participants were defined as CSII or continuous glucose monitoring (CGM) users when they were using the respective device for at least one visit during the observation period. The CGM category included both real-time CGM and intermittent CGM. SH and DKA were defined under the ISPAD Clinical Consensus Guidelines (16,17) [2] and pointed out as the proportion of episodes during the entire observation period.

Four groups were formed depending on the participants' insulin delivery method and the use of CGM. The groups were as follows: (1) MDI without CGM; (2) MDI with CGM; (3) CSII without CGM; (4) CSII with CGM (including both sensor-augmented pumps and hybrid closed loop systems). Each category was analyzed stratified by age group: 2.5–7 years (preschool), 7–<12 years (preadolescent), and 12–18 years (adolescent).

### 2.4. Statistical Analyses

Descriptive data were summarized using means with standard deviations (SD), medians with interquartile range (IQR), or proportion for binary variables. Wilcoxon test was used to reveal the differences between the two groups, while the Kruskal–Wallis test was used to compare more than two groups. Binary variables were compared by *χ*^2^ test. The *p* values were corrected using the Bonferroni-stepdown method to adjust for multiple testing.

Linear and fractional logistic regression models were used in order to adjust age (categorical), gender, diabetes duration (categorical), and treatment modality, and the methods were readjusted for multiple group comparisons using the Tukey–Kramer method. Results of linear regression models are presented as adjusted means with a 95% confidence interval (CI). Fractional logistic regression models were used to analyze BD/TDD adjusted for gender, age group, diabetes duration, and treatment modality.

Two-sided *p*-values of <0.05 were considered statistically significant. All analyses were performed with *SAS v.9.4* (*SAS Institute Inc*, *Cary*, *North Carolina*, *USA*).

## 3. Results

The study was conducted with a total of 38,956 individuals from a total of 122 centers in 57 countries. Among the sample, 48.6% were female, the median (IQR) age was 15.2 (11.9; 17.2) years, and the median (IQR) diabetes duration was 6.0 (3.8; 9.0) years. Treatment modality distribution was as follows; MDI without CGM, 32.9%; MDI with CGM, 18.0%; CSII without CGM, 11.7%; and CSII with CGM37.3%.  Table 1 summarizes the individual characteristics according to treatment modality.

### 3.1. Analysis of Treatment Modality

Data from each treatment modality was stratified by sex, age group, BMI-SDS, and HbA1c level was analyzed separately. While there was no significant difference between groups by sex, BMI-SDS, and HbA1c, it was observed that BD/TDD decreased with the use of technology only in the preadolescent group. The results are shown in Table 2.

### 3.2. Analysis Stratified by Treatment Models

Data of the participants were analyzed for each treatment modality separately. A significant association with BD/TDD was present for several variables, regardless of treatment modality. The significant variables were male gender, younger age group, and lower HbA1c, which were all related to lower BD/TDD (all *p* < 0.05). The results are shown in Table 3.

When grouped according to HbA1c levels, BD/TDD was significantly lower in participants with HbA1c on target, regardless of treatment modality, except for people with diabetes (PwD) on MDI with a CGM system. The lowest BD/TDD was in CSII with the CGM group (Table 3).

However, after the age, gender, and diabetes duration adjustment, no association was found between BD/TDD and the use of different diabetes technologies (CGM, CSII). According to treatment modality, mean (95% CI) BD/TDDs were 45.2 (44.4–46.1)% in MDI without CGM group, 44.6 (43.4–45.7)% in MDI with CGM group, 43.7 (42.2–45.1)% in CSII without CGM group, and 44.9 (44.1–45.7)% in CSII with CGM group (all *p* > 0.05).

## 4. Discussion

This study subjected the analysis of a large children and adolescents cohort with T1D and showed that treatment modality did not influence BD/TDD in both adjusted and nonadjusted comparisons. However, an association between lower BD/TDD and lower HbA1c or younger age or male gender was observed.

The use of pumps and CGM varies across healthcare providers internationally in pediatric populations. In addition, substantial differences have been observed in HbA1c outcomes. In our study, 49% of all PwD younger than 18 years old were using insulin pumps. Similarly, in a SWEET study, insulin pump use was 48% [9]. In the T1D Exchange (T1DX) Registry in the USA, 50% of young children were using insulin pumps compared to 74% in the Prospective Diabetes Follow-up Registry (DPV) in Germany and Austria [10]. In the present study, low BD/TDD was associated with lower HbA1c in all treatment modalities. In the HbA1c on the target group, children and adolescents using CSII with CGM had lower BD/TDD. The use of many daily boluses and a lower proportion of basal insulin revealed significantly better glycemic outcomes than using fewer boluses and a higher proportion of basal insulin, which was consistent with the findings of a previous CSII study that showed a significant correlation between missed mealtime boluses and elevated HbA1c [11].

Furthermore, the high BD/TDD impact on glycemic outcome has been reported in a number of studies. In a large study, including 1,098 PwD, Danne et al. [1] reported improved metabolic control in patients with BD/TDD below 50%. In another study, 78 children and adolescents with T1D with a follow-up period of 29 months reported that a 10% change in high BD/TDD resulted in a 0.22% change in HbA1c [3]. Often, basal insulin of 50% or more of the total daily dose combined with a high HbA1c may indicate nonadherence and missed insulin boluses or may indicate PwD or physicians increasing the basal insulin dose to cover missed boluses in children.

In our study cohort, BD/TDD was lower in individuals who were <7 years of age compared to the other age groups among all treatment modalities. Among age groups, different amounts of basal insulin were used overall. Compared to older children, toddlers have lower basal rates of insulin. The highest basal insulin dose, both in total and in relation to body weight, was reported in adolescents. Puberty is characterized by increased insulin resistance, which might explain the increase in weight-related basal insulin. Although the total daily insulin dose increases with puberty, basal insulin requirement rarely reaches adult levels of 50% or more, often ranging from 30% to 45% in children with good glycemic outcomes [12–14]. In a prospective database study subjecting children with T1D under pump therapy, basal insulin requirements were analyzed for individuals in three age groups. PwD children who were <6 years had the lowest basal requirement.

On the contrary, the basal requirement increased in older children, and the highest requirement was in children aged >12 years [5]. Similarly, Cemeroglu et al. [15] reported that the basal insulin requirement was much lower than the bolus requirement compared to adults with T1D, and the highest basal insulin requirement was 40%–45% of the total daily dose at the onset of puberty and the lowest 34% of the total daily dose in the first 7 years of life. Insulin requirements according to body weight were higher in the female gender than the male gender during puberty, consistent with previous reports [15, 16]. While the cause of the higher insulin resistance in adolescent girls with T1D compared to adolescent boys is unknown, it appears to be due to increased estrogen production in girls during puberty, which increases the risk for adiposity [17, 18].

An association between BD/TDD and BMI-SDS was not present in our study. Previous studies reported that insulin distribution was related to BMI [19–21]. The anabolic effect of insulin decreases lipolysis while stimulating protein synthesis and lipogenesis. A basal insulin replacement higher than the physiological needs may have an anabolic effect which leads to fat gain and BMI increase. In addition, a basal insulin replacement higher than physiological need may result in fasting hypoglycemia and, therefore, the need for extra carbohydrate intake.

Similarly, an individual receiving higher basal insulin may eventually need a carbohydrate snack at bedtime to avoid the risk of nocturnal hypoglycemia. Furthermore, few people who have T1D and have poor metabolic control have their actual basal insulin needs by eating a meal [22], and these individuals may require increased frequency and/or higher bolus doses rather than increased basal insulin. A study reported a positive correlation between BD/TDD and change in BMI during a 1-year period [23], whereas another study did not report such a correlation [24]. Rasmussen et al. [25] conducted a registry study and reported that a lower basal-to-total insulin ratio was associated with lower HbA1c and lower BMI-SDS in children who were under insulin pump therapy. The same study also reported that there was no association between BD/TDD and either HbA1c or BMI-SDS in individuals using MDI therapy. As a result, a low BD is related to metabolic control. PwD with a high basal dose deserves more attention and multidisciplinary intervention.

## 5. Conclusion

In summary, we describe an association between a lower HbA1c and a low basal distribution concerning the total daily dose in a large and diverse cohort of children with T1D. Lower basal insulin was related to a younger age. However, due to the large sample size, even small differences can be classified as statistically different, and the clinical relevance is arguable. Moreover, no association was found between BD/TDD and treatment modalities. Longitudinal studies are needed to assess the impact of basal/bolus insulin distribution on glycemic outcome and body composition in children and adolescents with T1D.

## Figures and Tables

**Table 1:** Characteristics of the cohort.

	MDI without CGM (*n* = 12,822)	MDI with CGM (*n* = 7,020)	CSII without CGM (*n* = 4,569)	CSII with CGM (*n* = 14,545)	*p* Values
Mean age (years)	14.5 ± 3.4	13.8 ± 3.4	15.1 ± 3.3	13.7 ± 3.6	*p* ^1^
Mean age at diabetes onset (years)	8.1 ± 3.9	7.9 ± 3.8	7.3 ± 3.9	6.7 ± 3.7	<0.05
Mean diabetes duration (years)	6.5 ± 3.5	5.9 ± 3.3	7.8 ± 3.7	7.0 ± 3.5	<0.05
Female/male (%)	48.4/51.6	47.4/52.6	50.4/49.6	48.8/51.2	*p* ^2^
Mean height-SDS	−0.08 ± 1.08	0.10 ± 1.14	0.38 ± 1.00	0.43 ± 0.99	<0.05
Mean BMI-SDS	−0.05 ± 1.13	0.06 ± 1.10	0.18 ± 0.97	0.28 ± 0.96	<0.05
Mean insulin dose (IU/kg)	0.97 ± 0.33	0.96 ± 0.36	0.82 ± 0.22	0.82 ± 0.24	*p* ^3^
≥1 severe hypoglycemia (%)	4.5	4.3	3.4	2.5	*p* ^4^
≥1 diabetic ketoacidosis (%)	3.2	1.9	2.5	1.3	*p* ^5^

*p* ^1^: <0.001 for 1 vs. 2, 1 vs. 3, 2 vs. 3, 1 vs. 4, 3 vs. 4, *p*^2^: <0.001 for 2 vs. 3, *p*^3^: <0.001 for 1 vs. 2, 1 vs. 3, 2 vs. 3, 1 vs. 4, 2 vs. 4, *p*^4^: <0.05 for 1 vs. 3, 1 vs. 4, 2 vs. 4, *p*^5^: <0.05 for 1 vs. 2, 1 vs. 4, 2 vs. 4, 3 vs. 4.

**Table 2:** Basal dose/total daily dose ratios (%) in treatment modality (unadjusted).

Groups	MDI without CGM (%) (1)	MDI with CGM (%) (2)	CSII without CGM (%) (3)	CSII with CGM (%) (4)	*p* Values
Gender					
Female	46 ± 14	45 ± 12	45 ± 14	46 ± 12	*p* ^1^
Male	45 ± 14	44 ± 12	43 ± 13	44 ± 12	*p* ^2^
Age (year)					
<7	43 ± 14	43 ± 14	41 ± 13	40 ± 12	*p* ^3^
7–11	45 ± 13	44 ± 12	42 ± 14	42 ± 12	<0.05
12–18	45 ± 14	44 ± 12	45 ± 14	46 ± 12	*p* ^4^
BMI SDS					
≤−2	47 ± 15	45 ± 12	45 ± 17	43 ± 14	*p* ^5^
−1.9 to ≤1	46 ± 14	44 ± 12	44 ± 14	44 ± 12	*p* ^6^
1 to ≤2	45 ± 13	44 ± 11	42 ± 12	43 ± 12	*p* ^7^
>2	47 ± 13	45 ± 13	46 ± 13	44 ± 12	*p* ^8^
HbA1c					
≤7.5%	44 ± 14	44 ± 13	44 ± 13	43 ± 12	*p* ^8^
7.6%–9%	45 ± 13	44 ± 12	43 ± 13	46 ± 12	*p* ^9^
>9%	46 ± 14	45 ± 11	47 ± 16	50 ± 13	*p* ^10^

*p* ^1^: <0.001 for 1 vs. 4, 2 vs. 4, 3 vs. 4, *p*^2^: <0.001 for 1 vs. 2, 1 vs. 3, 2 vs. 3, 1 vs. 4, 3 vs. 4, *p*^3^: <0.001 for 1 vs. 4, 2 vs. 4, *p*^4^: <0.05 for 1 vs. 2, 1 vs. 3, 1 vs. 4, 2 vs. 4, 3 vs. 4, *p*^5^: 0.01 for 1 vs. 4, *p*^6^: <0.001 for 1 vs. 2, 1 vs. 3, 1 vs. 4, *p*^7^: <0.001 for 1 vs. 3, 2 vs. 3, 1 vs. 4, *p*^8^: <0.05 for 1 vs. 4, *p*^9^: <0.001 for 1 vs. 3, 2 vs. 3, 1 vs. 4, 2 vs. 4, 3 vs. 4, *p*^10^: <0.001 for 2 vs. 3, 1 vs. 4, 2 vs. 4, 3 vs. 4. MDI, multiple daily injections; CSII, subcutaneous insulin infusion system; CGM, continuous glucose monitoring; BMI, body mass index; HbA1c, hemoglobin A1c.

**Table 3:** Basal dose/total daily dose ratios (%) in age, BMI-SDS, and HbA1c (unadjusted).

	Age (year)				BMI SDS					HbA1c (%)			
	<7 (1)	7–11 (2)	12–18 (3)	*p*	≤−2 (1)	−1.9 to ≤1 (2)	1 to ≤2 (3)	>2 (4)	*p*	≤7.5 (1)	7.6–9 (2)	>9 (3)	*p*
MDI without CGM (*n*: 16,177)	43 ± 14	45 ± 13	45 ± 14	*p* ^1^	47 ± 15	46 ± 14	45 ± 13	47 ± 13	>0.05	44 ± 14	45 ± 13	46 ± 14	<0.001
MDI with CGM (*n*: 10,018)	43 ± 14	44 ± 12	44 ± 12	>0.05	45 ± 12	44 ± 12	44 ± 11	45 ± 13	*p* ^2^	44 ± 13	44 ± 12	45 ± 11	*p* ^1^
CSII without CGM (*n*: 7,513)	41 ± 13	42 ± 14	45 ± 14	*p* ^3^	45 ± 17	44 ± 14	42 ± 12	46 ± 13	>0.05	44 ± 13	43 ± 13	47 ± 16	<0.001
CSII with CGM (*n*: 13,868)	40 ± 12	42 ± 12	46 ± 12	<0.00	43 ± 14	44 ± 12	43 ± 12	44 ± 12	>0.05	43 ± 12	46 ± 12	50 ± 13	<0.001

*p* ^1^: <0.001 for 1 vs. 2, 1 vs. 3, *p*^2^: 0.027 for 1 vs. 2, *p*^3^: <0.001 for 1 vs. 3, 2 vs. 3. MDI, multiple daily injections; CSII, subcutaneous insulin infusion system; CGM, continuous glucose monitoring; BMI, body mass index; HbA1c, hemoglobin A1c.

## Data Availability

The data used to support the findings of this study are available from the corresponding author upon request.

## Additional Points

*Strengths and Limitations*. The major strengths of this study are that it provides real-world data from a large number of clinics across multiple regions, assuming diversity in ethnicity, eating patterns, nutrition, and physical activity. The limitation is that hybrid closed-loop systems were included in the larger group of CSII, which could have potentially skewed data as hybrid closed-loop systems automatically adjust the basal rates up and down to accommodate for variability in blood glucose because the sample size was inadequate to establish comparisons as a single group. Moreover, additional adjustments for the center region yielded similar results (data not shown). BD/TDD ratio could be different if the staple food is a carbohydrate, such as in Asian countries. BD/TDD ratio is lower than in Western countries in these countries. We did not adjust for the region; only gender and diabetes duration were adjusted, as mentioned in the paper. HbA1c was not an outcome variable, so we did not adjust HBA1c for the region. However, models adjusting for the region were calculated, and similar results in the regression models were found (data not shown).
